# Microstructure Evolution of Graphene and the Corresponding Effect on the Mechanical/Electrical Properties of Graphene/Cu Composite during Rolling Treatment

**DOI:** 10.3390/ma15031218

**Published:** 2022-02-06

**Authors:** Ziyang Xiu, Boyu Ju, Junhai Zhan, Ningbo Zhang, Zhijun Wang, Yong Mei, Jinming Liu, Yuhan Feng, Yixin Guo, Pengchao Kang, Qiang Zhang, Wenshu Yang

**Affiliations:** 1 State Key Laboratory of Advanced Welding and Joining, Harbin Institute of Technology, Harbin 150001, China; xiuzy@hit.edu.cn; 2 School of Materials Science and Engineering, Harbin Institute of Technology, Harbin 150001, China; hitzhijun@gmail.com (Z.W.); gyx082339@icloud.com (Y.G.); 3 Shanghai Aerospace System Engineering Research Institute, Shanghai 201108, China; JPY_mat@163.com; 4 Aerospace Research Institute of Materials & Processing Technology, Beijing 100076, China; zhangnbathit@163.com; 5 School of Astronautics, Harbin Institute of Technology, Harbin 150001, China; 6 Defense Engineering of Academy of Military Sciences, PLA Academy of Military Sciences, Beijing 100036, China

**Keywords:** graphene/cu composite, rolling treatment, graphene dispersion, graphene defects

## Abstract

Rolling enables the directional alignment of the reinforcements in graphene/Cu composites while achieving uniform graphene dispersion and matrix grain refinement. This is expected to achieve a breakthrough in composite performance. In this paper, the process parameters of rolling are investigated, and the defects, thickness variations of graphene and property changes of the composite under different parameters are analyzed. High-temperature rolling is beneficial to avoid the damage of graphene during rolling, and the prepared composites have higher electrical conductivity. The properties of graphene were investigated. Low-temperature rolling is more favorable to the thinning and dispersion of graphene; meanwhile, the relative density of the composites is higher in the low-temperature rolling process. With the increase of rolling deformation, the graphene defects slightly increased and the number of layers decreased. In this paper, the defect states of graphene and the electrical conductivity with different rolling parameters is comprehensively investigated to provide a reference for the rolling process of graphene/copper composites with different demands.

## 1. Introduction

Cu metals, alloys, and composites are widely used in industrial production. Cu has excellent electrical and thermal conductivity, as well as good processability and corrosion resistance, second only to Ag [1,2]. They play a vital role in key fields such as electronic packaging, microelectronics industry, national defense, and aerospace [3,4,5]. Graphene is an emerging reinforcement material. Due to the unique two-dimensional structure of graphene, it has ultra-high thermal conductivity (up to 5300 W/(mK)) and excellent mechanical properties [6,7], and has been made a good selection for different industrial applications [8,9,10]. Graphene nanoplates (GNPs) are used as reinforcements and added to the pure copper metal matrix to prepare GNPs/Cu composites. Theoretically, they can retain the high electrical conductivity and high thermal conductivity of the Cu metal material while having good mechanical properties [11,12]. Moreover, graphene/Cu composite has good designability and is expected to be applied in the fields of different structures and functional materials [13,14,15].

However, the current development of graphene/Cu composite faces the following problems. Since graphene is a nano reinforcement, it is very easy to agglomerate after being prepared into a composite, which causes the two-dimensional strengthening characteristics of the graphene to fail to fully play, which reduces the performance [16,17]. In addition, graphene is easily damaged during material processing [18,19]. The defect causes a substantial decrease in the electrical conductivity of graphene.

The uniform dispersion of graphene in the metal matrix can be achieved through the deformation of the composites. Shao et al. [20,21] found that the hot extrusion process can make the graphene oriented in the Al matrix. Under the action of shearing, the graphene was also twisted, bent, and opened by shearing between layers, so that the graphene was further dispersed. The obtained composite had a tensile strength of 500 MPa, which was 40% higher than the performance of the Al matrix. Wu et al. [22] studied the effect of multi-pass extrusion on graphene/6063Al composites. It is found that after multiple extrusions, graphene had an obvious interlayer slip, and fewer graphene nanoplates were peeled off from the thick graphene layer. After 3-cycle extrusion, the plasticity of GNPs/6063Al composites increased from 7% to 13% without any decrease in strength. The improvement of composite performance comes from the fact that graphene is less layered and redispersed after multiple extrusions so that the strengthening ability of graphene can be further exerted. Huang et al. [23] studied the properties of rolled graphene/Al composites at different temperatures and found that when the temperature is below 500 °C, the tensile strength of the material gradually increases as the temperature rises. When the rolling temperature exceeded 500 °C, the mechanical properties of the material decreased as the temperature increased, and a large amount of reaction product Al_4_C_3_ was observed at the interface.

Wang et al. [24] reported the peeling behavior of graphene during the rolling process. It was found that the graphene structure evolved according to the following process: firstly, it was aligned along the rolling direction, then broken into small pieces, and finally exfoliated into a few layers of graphene. The exfoliated graphene provided an additional contribution to the strength and plasticity of the composite. Korznikova et al. [25] studied the graphene/Cu composites consolidation by means of its high-pressure torsion (HPT). It was found that under the action of deformation, the grains of the Cu matrix were refined, and the graphene was more uniformly dispersed. The elongation of the composite decreased from 15% to 4% after deformation, but the hardness increased by almost 30%. Yao et al. [26] investigated the variation of Cu/C composites during accumulative roll-bonding (ABR). Results showed that ARB can remarkably decrease the size of graphite and improve the dispersion of graphite in the Cu matrix. Tiwari et al. [27] investigated graphene/Al composites prepared by ARB and found that the tensile strength and hardness of the composites were enhanced by 25% and 20%, respectively, which was attributed to the fraction of high angle grain boundaries enhancement and homogeneous dispersion of graphene. Liu et al. [28] investigated the organization of Graphene nanosheets/Cu composites after ARB treatment and found the average spacing of high-angle boundaries along the normal direction was finer than that of the ARBed Cu. In addition, high-density deformation-twins with grain sizes of 50–70 nm are present at the interface.

The parameters of the deformation have an important influence on the dispersion and deformation of graphene in the matrix, but the rules are still unclear. However, the research on the specific effects of deformation temperature and deformation on the structure and properties of the composite is not comprehensive enough.

In this work, the microstructural evolution behavior of graphene under different rolling parameters was studied. Due to the stable chemical properties of Cu and no interfacial reaction with graphene, it was convenient to observe the deformation behavior of graphene. Therefore, Cu was selected as the matrix for preparing composites. In order to clarify the deformation behavior of graphene in the matrix, this paper comprehensively studied the effects of deformation temperature and rolling deformation on graphene/Cu composites. The organization evolution of graphene was studied through Raman spectroscopy, and the mechanical and electrical properties were tested, which provide a reference for subsequent deformation of composite materials.

## 2. Materials and Methods

**Figure 1:**
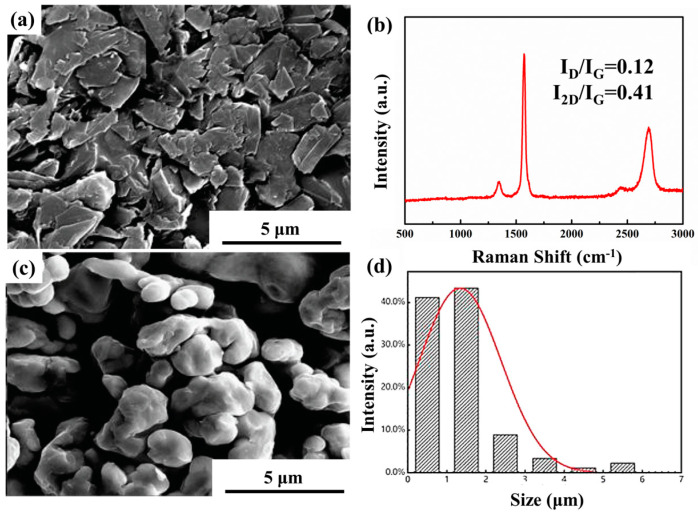
Microstructure characterization of graphene and Cu. (**a**) SEM of graphene, (**b**) Raman characterization of graphene, (**c**) SEM of Cu powders, (**d**) particle size distribution of Cu powders.

The graphene used in this experiment was supplied by the Sixth Element Changzhou Materials Technology Co. Ltd. China. The pure Cu powder used in this study was purchased from Beijing Xingrongyuan Co., Ltd., Beijing, China, with a purity of 99.9% and an initial particle size of about 3 μm. The characterization of graphene and Cu powder is shown in Figure 1. Figure 1a,b show the SEM and Raman characterization of graphene, respectively. Graphene is distributed in flakes. In the Raman characterization, there are mainly three characteristic peaks, namely the D peak at 1350 cm^−1^, the G peak at 1570 cm^−1^, and the 2D peak at 2700 cm^−1^. The D peak reflects the asymmetric lattice vibrations, and the G peak reflects the symmetrical lattice vibrations [29,30]. The intensity ratio of the D peak and the G peak (I_D_/I_G_) is often used to reflect the change of graphene defects. 2D peaks appearing at approximately 2700 cm^−1^ is related to the number of graphene layers. And the peak position, peak shape, and intensity of 2D peaks can be used to judge the number of graphene layers [31,32,33]. The smaller the number of graphene layers, the higher the intensity of the 2D peaks and the shift toward lower wavenumbers [34,35].

In this study, a planetary ball mill (YXQM-4L, supplied by Miqi Equipment Co., Ltd., Changsha, China) was used to disperse the graphene, and the ball-milling speed of 120 r/min for 2 h was used. The content of graphene is 0.6 wt.%. The mixed graphene-Cu mixed powder is sintered through SPS. The powder mixture of GNPs and Cu was put into a 40 mm diameter mold and pressed into preforms at a pressure of 40 MPa. The pressure was maintained for 5 min. The mold with the preforms was then placed into the SPS equipment and heated to 1020 °C at 5 °C/min and kept at high temperature for 10 min. The current-to-time ratio (ton:toff) during sintering was 2:1. After the sintering was completed, the sample was cooled to room temperature in the mold with the furnace. The prepared 0.6 wt.% GNPs/Cu composite was subjected to subsequent rolling research.

In this work, a double-roll mill (supplied by Sanye Mould Co., Ltd., Taizhou, China) is used to optimize the deformation of the prepared composite. Under the action of the stresses generated during the rolling process, the Cu matrix and graphene undergo shear deformation. The total thickness reduction (%) is counted as the amount of down-compression deformation. The 0.6 wt.% graphene/Cu composite material was cut into 55 mm × 10 mm × 4 mm rectangular parallelepiped and rolled from the original thickness of 4 mm. The prepared composite is rolled at 20 °C, 300 °C, 500 °C, 700 °C. The deformation amount of each rolling is 10%, and the samples with large deformation amounts are prepared by the method of multiple rolling. The total reduction of multiple rolling is 20–80%. The hardness and electrical conductivity of the composites were subsequently investigated at different rolling temperatures and deformation amounts.

Morphologies of the GNPs/Cu powders and the composites were observed by FEI Quanta 200FEG (supplied by Thermo Fisher Scientific Co., Ltd., Bend, OR, US). Raman analysis was performed on a JY-HR800 laser Raman spectrometer (supplied by HORIBA Ltd., Montpellier, France) using a 532 nm solid-state laser as an excitation source. The image analysis microhardness of HV-1000IS (provided by Jujing Precision Instrument Manufacturing Co., Ltd. Shanghai, China) was used to test the Vickers hardness of the graphene/Cu composites. In the hardness test, the experimental parameter load is 200 gf, the pressure time is 15 s. An FD 102 digital eddy current conductivity meter (supplied by Foster Electronic Technology Co., Ltd., Xiamen, China) was used for the conductivity test. Each sample is randomly measured 5 times at a certain distance, and the average value is obtained. The density of the material was measured by Archimedes’ drainage method. The mass of the sample in air and the mass in water are tested separately and the density of the material is calculated according to the formula:

$$\rho_{m}{=\rho }_{H2O}\frac{m_{1}}{m_{1}{-m}_{2}}$$

where *ρ_m_* and *ρ_H_*_2_*_O_* are the densities of the composite and the sample, respectively, and *m*_1_ and *m*_2_ are the masses of the composite in air and water, respectively. The relative density is the ratio of the experimental density to the theoretical density of the sample.

## 3. Results and Discussion

### 3.1. The Effect of Rolling Temperature on the Structure of Graphene

**Figure 2:**
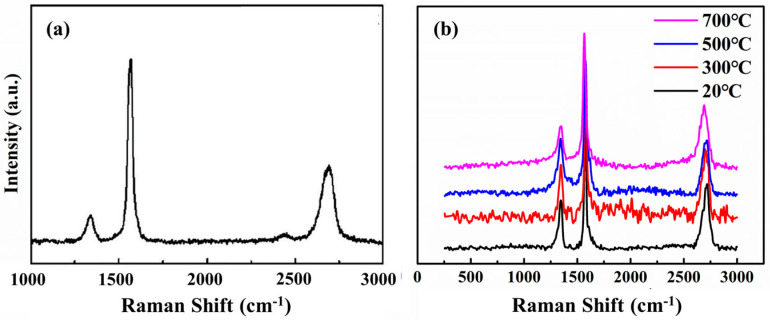
Raman characterization of 0.6 wt.% graphene/Cu composites before and after rolling at different temperature. (**a**) Composite before rolling, (**b**) Composite rolled at different temperature.

The GNPs/Cu composite material was rolled and deformed at rolling temperatures of 20 °C, 300 °C, 500 °C, and 700 °C, and the total deformation was 80%. Raman characterization of the composite material, the results are shown in Figure 2. The I_D_/I_G_ of graphene in the composite before rolling is 0.20, and the I_2D_/I_G_ is 0.43. The state of the graphene in the sintered composite is close to that of the raw graphene because the graphene is less damaged during the preparation processing. The defect content of graphene only increased slightly, and the number of layers did not change significantly.

**Figure 3:**
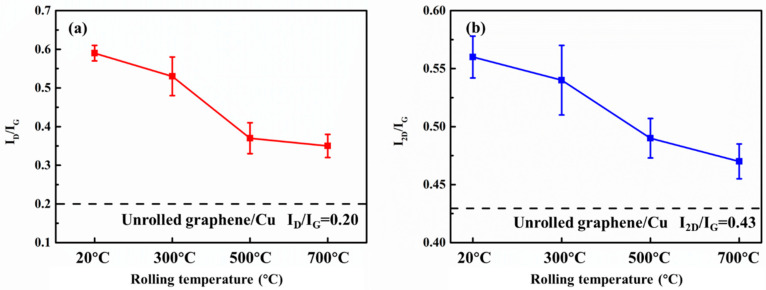
Quantitative statistics of Raman peak intensity of rolled composites at different temperatures. (**a**) I_D_/I_G_, (**b**) I_2D_/I_G_.

After rolling at different temperatures, the graphene changes significantly. Comparing the positions of the characteristic peaks of the Raman spectrum curve at different rolling temperatures, it can be seen that the positions of the D peak and the G peak are basically the same. The peak shape of the 2D peak changes slightly due to the change in the number of graphene layers. The ratio of the intensity of change of the graphene D peak, the G peak, and the 2D peak is quantified, as shown in Figure 3.

It can be seen that when the composites were rolled, the I_D_/I_G_ of graphene increased significantly compared to the unrolled graphene/Cu composites, indicating a significant increase in the defect content in graphene. Graphene suffers huge damage during the rolling process. I_2D_/I_G_ also increased slightly, indicating that the number of graphene layers decreased during the rolling process.

**Figure 4:**
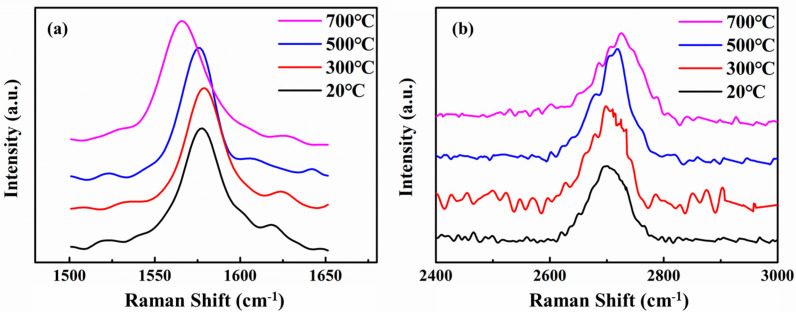
The peak shapes of G peak and 2D peak under different rolling temperatures. (**a**) G peaks, (**b**) 2D peaks.

As the rolling temperature rises, I_D_/I_G_ and I_2D_/I_G_ drops slightly. It shows that after the rolling temperature is increased, the rolling damage to the graphene is less, and at the same time, the peeling effect of the graphene is reduced. This is because when the rolling temperature is lower, the composite is subjected to greater stress during cold rolling. Graphene shears, bends, and twists as the substrate deforms, creating more edge and hole defects. At the same time, the large shear stress also causes the multi-layer graphene layer to slip, which reduces the number of graphene layers. To further characterize the changes in the number of graphene layers at different rolling temperatures, the deviation of the graphene G peak and 2D peak is observed, as shown in the Figure 4.

Based on the research results of Malard et al. [33], as the number of graphene layers decreases, the 2D peak wave number decreases. If the G peak is used as a reference, the greater the wavenumber difference between the 2D peak and the G peak, the greater the number of graphene layers. It can be seen from Figure 4 that as the rolling temperature decreases, the G peak shifts to the right and the 2D peak shifts to the left. As the rolling temperature decreases, the number of graphene layers decreases. The lower the rolling temperature, the more obvious the graphene thinning. The D peak position of the composite rolled at 20 °C is 2696 cm^−1^. Based on the results reported in other papers [32,33], it can be seen that the number of graphene layers has dropped below 3 layers. It shows that rolling has a significant shearing and exfoliation effect on graphene, and has an important contribution to the reduction of graphene in the Cu matrix.

### 3.2. The Influence of Rolling Deformation on Structure of Graphene

**Figure 5:**
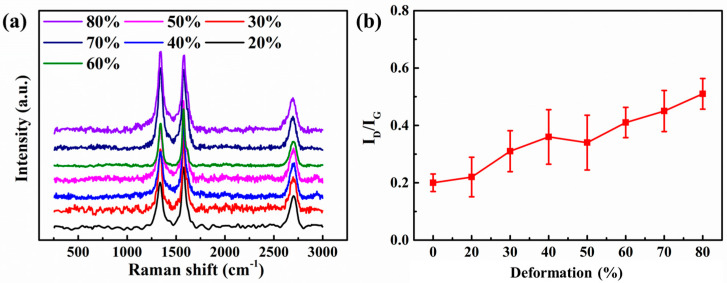
Raman characterization of 0.6 wt.% graphene/Cu composites at different rolling deformation. (**a**) Results of Raman characterization, (**b**) Quantitative statistics of I_D_/I_G_ peak intensity.

At 700 °C, under the condition of 10% deformation in a single press, the composite is subjected to multiple rolling treatments. The total rolling deformation is 4.0 mm → 10% (3.6 mm) → 20% (3.2 mm) → 30% (2.8 mm) → 60% (2.4 mm) → 50% (2.0 mm) → 60 (1.6 mm) → 70% (1.2 mm) → 80% (0.8 mm). The Raman characterization of rolled composites with different deformations is performed, and the results are shown in Figure 5a,b.

The intensity ratio of the D peak and the G peak is quantitatively calculated to determine the state of graphene defects, as shown in Figure 5b. The I_D_/I_G_ ratio of different total deformations has shown an upward trend from 20% to 80%, indicating that the defects of graphene are gradually increasing during the rolling process. At the beginning of rolling, the value of I_D_/I_G_ has a large standard deviation value, indicating that the structure is not uniform in the initial stage of deformation. When the total deformation is 40%, the standard deviation values of the I_D_/I_G_ ratio begin to decrease, indicating that the change of graphene begins to stabilize. As the rolling deformation continues, when the total deformation reaches 70%, the I_D_/I_G_ ratio once again greatly increases. The graphene defects increase significantly, and the variance of the I_D_/I_G_ ratio continues to increase. It showed that in the later stage of rolling deformation, under the action of large deformation, the Cu matrix began to crack, and stress concentration was formed at the crack during the deformation, which caused more serious damage to the graphene near the crack.

**Figure 6:**
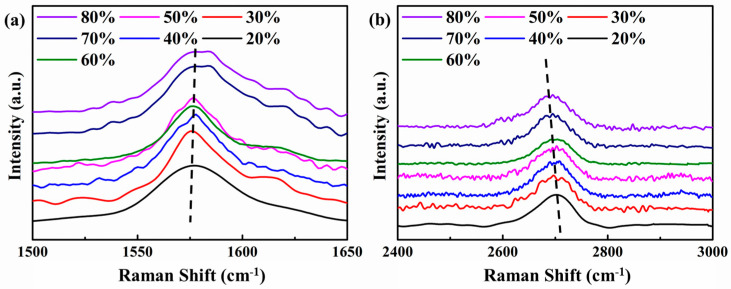
The peak shapes of G peak and 2D peak under different rolling deformation. (**a**) G peaks, (**b**) 2D peaks.

The shift of graphene G and 2D peaks in rolled composites with different deformations was observed, as shown in Figure 6. It can be seen that as the number of deformations increases, the position of the G peak does not change significantly, while the 2D peak shifts to the low wavenumber direction. It can be seen from the results that as the amount of rolling deformation increases, the number of graphene layers decreases. The reason for graphene thinning is the same as the previous study. As the matrix deforms, graphene slips between layers and the number of layers decreases.

### 3.3. Relative Density and Microstructure of Composites Obtained under Different Rolling Parameters

To further study the change law of the composite microstructure and explain the abnormal rise of graphene defects after the deformation exceeds 70% during cold rolling, the relative density and microstructure of the composite are characterized.

**Figure 7:**
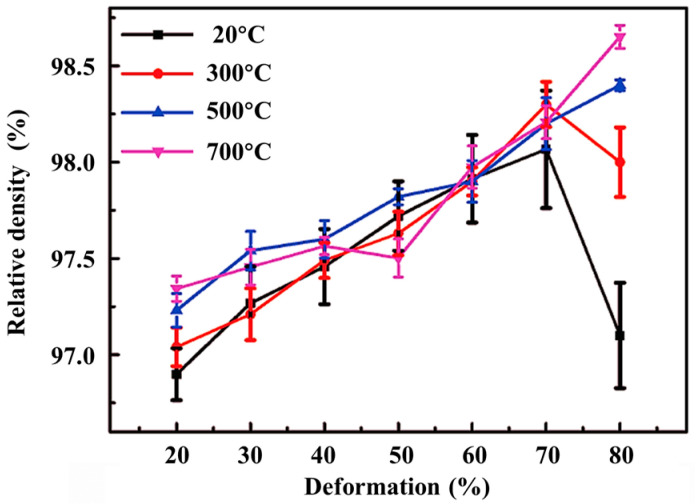
Relative density of composites rolled at different temperatures and deformations.

The relative density of the composite rolled at different temperatures and different deformation amounts are shown in Figure 7. It can be seen that there are differences in the relative density of composites rolled at different temperatures. When the rolling temperature is higher (500–700 °C), the relative density of the composite is slightly higher than that of low-temperature rolling (20–300 °C). This is because the Cu matrix is more prone to deformation under high-temperature conditions. And the defects such as pores and cracks are eliminated during the rolling process, increasing the density of composites. With the increase in rolling deformation, the relative density is on the rise. When the deformation exceeds 70%, the density of the low-temperature rolled composite will be greatly attenuated, while the density of the high-temperature rolled composite material will increase slightly.

**Figure 8:**
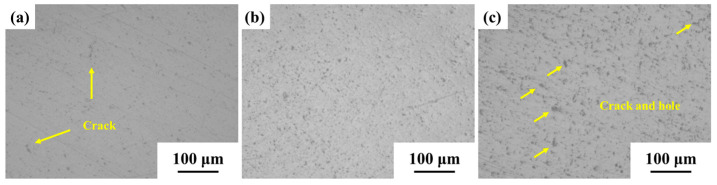
SEM characterization of 0.6 wt.% graphene/Cu composites under different deformations. (**a**) 30%, (**b**) 50%, (**c**) 80%.

SEM observation is performed on the 0.6 wt.% graphene/Cu composite rolled at 20 °C, and the reason for the decrease in density is analyzed, as shown in Figure 8. Figure 8a is a sample with 30% deformation. It can be seen that there are a few cracks and holes, as shown by the yellow arrow. As the rolling amount increases, the cracks gradually disappear, and the relative density increases. Figure 8c shows a sample with 80% deformation. When the deformation is too large, a large number of cracks and holes reappear in the material. When the rolling temperature is low, dislocations do not move easily, resulting in a Cu matrix that is not easily deformed. At higher rolling deformation, the matrix will directly crack and produce pores, resulting in a significant decrease in the density of the composite.

### 3.4. Hardness and Electrical Properties of Composites Obtained under Different Rolling Parameters

**Figure 9:**
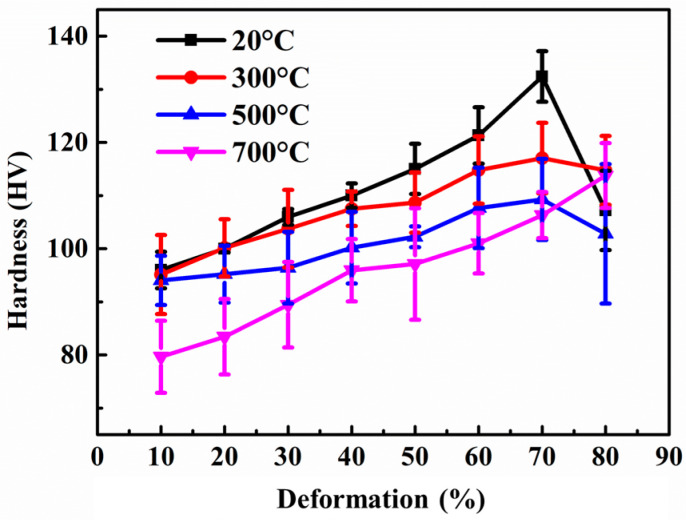
Hardness characterization of 0.6 wt.% graphene/Cu composites under different temperatures and deformations.

The hardness test of the composite after rolling treatment at different temperatures and deformations is shown in Figure 9. It can be seen that the composite obtained by rolling at 20 °C has the highest hardness. As the deformation increases, the hardness increases from 95 HV to 131 HV. When the amount of deformation reaches 80%, the hardness drops significantly to 105 HV. The hardness of the composite rolled at 700 °C is lower than 20 °C. As the amount of deformation increases, the hardness increases from 81 HV to 110 HV, and the hardness has not decreased when the deformation exceeds 80%.

Combining the density and the Raman characterization, the reasons for the performance changes can be analyzed. When the amount of rolling deformation is low, as the amount of deformation increases, the agglomerated multilayer graphene is thinned, and the matrix defects are reduced, increasing the hardness of the composite. When the deformation exceeds 70%, the Cu matrix will crack and the graphene will be severely broken, which will degrade the properties of composites. The performance after rolling shows an overall downward trend as the temperature increases. Combined with the Raman characterization, it can be seen that when the rolling temperature is lower, the matrix has greater stress, so the graphene has a more obvious thinning effect. At the same time, the graphene is more uniformly dispersed, and the strengthening ability is more easily exerted. Therefore, the material has greater hardness when rolled at low-temperature. The change in the properties of the composites was also related to the change in Cu grain size. It was reported that the Cu matrix grains were found to be coarse at higher rolling temperatures and the material properties decreased substantially [36,37,38]. In addition, the degree of graphene dispersion is higher and more uniform at low temperature rolling. Fei [39,40,41] and Zhao [14,42] et al. found that graphene has a hindering effect on the movement of dislocations in the metal matrix. When the degree of graphene dispersion is higher, the locking effect on dislocations is more obvious, which also contributes more to the hardness enhancement of the material.

**Figure 10:**
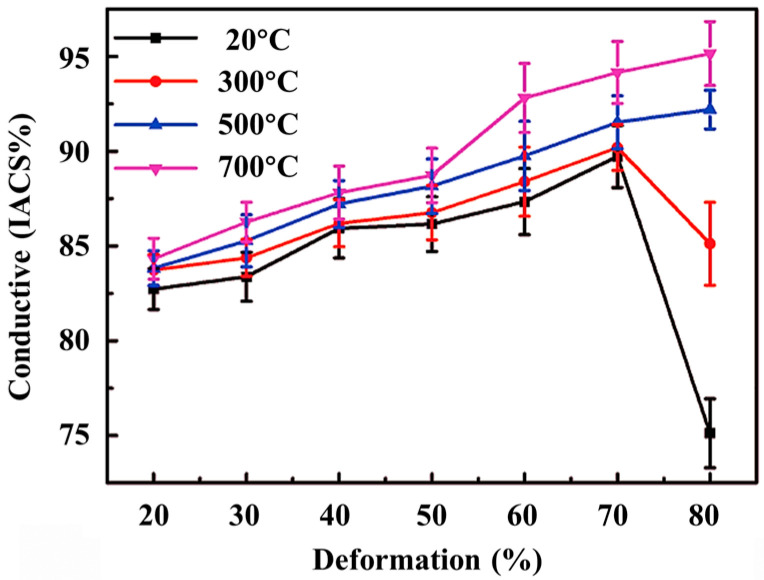
Conductive characterization of 0.6 wt.% graphene/Cu composites under different temperatures and deformations.

Afterward, the electrical conductivity of the composites rolled at different temperatures and deformations is tested, and the results are shown in Figure 10. The measured conductivity result is expressed by IACS (International Annealed Copper Standard). It can be seen that the change law of conductivity is opposite to that of hardness. When rolled at 700 °C, the rolled composite has a higher electrical conductivity. As the amount of deformation increases, the electrical conductivity increases from 83.7 IACS% to 95.2 IACS%. The conductivity of the sample rolled at 20 °C is lower, increasing from 82.5 IACS% to 89.6 IACS%. When the deformation reaches 80%, the electrical conductivity of the low-temperature rolled samples also appears to be greatly reduced, which is related to the reduction of density and graphene fragmentation.

As the rolling temperature increases, the conductivity of the composite increases. Based on previous studies, it can be seen that the higher the rolling temperature, the less damage the rolling will cause to graphene. Low-damage graphene has more contribution to electrical conductivity, so the electrical conductivity of composites prepared by high-temperature rolling is higher. The conductivity is also related to the grain size of the Cu matrix, the larger the grain size and the less grain boundary content, the higher the conductivity [43]. The grain size of the Cu matrix is larger during high temperature rolling and therefore also contributes positively to the electrical conductivity of the material.

In addition, rolling can produce an oriented arrangement of graphene in the composite. Guo et al. [44] investigated the properties of directionally aligned graphene/Al8030 composites and found that the yield strength, tensile strength, and electrical conductivity of the materials were substantially increased in the direction of the directional alignment of graphene. When the graphene/Al composites fracture, microcracks preferentially arise from the weak interface between the GNPs and the matrix. Then the cracks extend into the grains and deflect when they encounter the next GNPs. Finally, the cracks gradually extend until fracture. Directionally aligned graphene can create more obstacles for crack extension, which greatly enhances the path of crack movement when the material fractures and is an important reason for the material to maintain plasticity and increase strength.

## 4. Conclusions

In this work, the microstructure and properties under different rolling temperatures and rolling deformation were systematically investigated. When the rolling temperature decreases and the deformation increases, the thinning effect of graphene during rolling is enhanced, and more damage to graphene is also produced. At rolling temperatures below 300 °C, rolling facilitates the uniform dispersion of few-layer graphene in the Cu matrix. When the temperature is higher than 300 °C, the damaging effect of rolling on graphene can be reduced.

The hardness and electrical conductivity of the composites increased with the increase of rolling deformation. When the deformation of low-temperature rolling exceeds 70%, its properties are weakened. The hardness of the composites decreases with increasing rolling temperature, while the electrical conductivity increases with increasing rolling temperature. In this paper, the structural and property changes of the composites under different rolling processes are comprehensively studied to provide a reference for the rolling process of graphene/copper composites with different demands.

## Data Availability

The data presented in this study are available on request from the corresponding author.
